# Timing of onset of symptom for COVID-19 from publicly reported confirmed cases in Uganda

**DOI:** 10.11604/pamj.2021.38.168.27673

**Published:** 2021-02-15

**Authors:** Alex Riolexus Ario, Bernadette Basuta Mirembe, Claire Biribawa, Lilian Bulage, Daniel Kadobera, Robert Wamala

**Affiliations:** 1 Ministry of Health, Kampala, Uganda,; 2 Uganda National Institute of Public Health, Kampala, Uganda,; 3 African Field Epidemiology Network, Kampala, Uganda,; 4 Directorate of Research and Graduate Training, Makerere University, Kampala, Uganda

**Keywords:** COVID-19, SARS-CoV-2, incubation period, Uganda

## Abstract

**Introduction:**

incubation period for COVID-19, 2-14 (average 5-6) days. Timing of onset of COVID-19 signs and symptoms amongst cases in Uganda is however not known.

**Methods:**

we utilized data on real-time reverse transcription polymerase chain reaction (RT-PCR) confirmed cases to investigate symptom onset timing, from 21^st^ March to 4^th^ September 2020. Since timing of COVID-19 symptom onset is highly likely to be an interval rather than a point estimate, we generated 3-tertile categories: 1^st^, 2^nd^ and 3^rd^ tertile denoting symptom presentation within 3, 4 to 6 and at least 7 days. We considered all signs and symptoms in the database and analysed using Chi-square test and multinomial logistic regression, controlling for age and sex.

**Results:**

we analysed a total of 420 symptomatic case-patients; 72.0% were males, median age of 33 years. Common symptoms were cough (47.6%), running nose (46.2%), fever (27.4%), headache (26.4%) and sore throat (20.5%). We utilized 293 cases with clinical symptom onset date recorded. Most of the patients, 37.5%, presented symptom within 3 days, 31.4% had symptoms in the 2^nd^ and 31.4% in 3^rd^ tertile, denoting 4 to 6 days and at least 7 days after exposure. Running nose (RRR=0.45, 95%CI: 0.24-0.84) and chest pain (RRR=0.64, 95%CI: 0.09-0.72) were more likely to occur in 3^rd^ tertile than 1^st^ or 2^nd^ tertile. Cases aged ≥20 years were less likely to have symptoms in the 1^st^ and 2^nd^ tertile compared to ≤20 years (p<0.05).

**Conclusion:**

our study provides empirical evidence for epidemiological characterization of cases by signs and symptoms which complements current proposals for the length of active monitoring of persons exposed to SARS-CoV-2.

## Introduction

The outbreak of the novel coronavirus disease (COVID-19) caused by severe acute respiratory syndrome coronavirus (SARS-CoV-2) was first reported in Wuhan City, China in December 2019 but quickly spread across the world by travelers who originated from or passed through COVID-19 hotspots. On 30^th^ January 2020, the World Health Organization (WHO) declared COVID-19 outbreak a public health emergency of international concern (PHEIC) [1,2]. Shortly afterwards, COVID-19 was declared a pandemic. Africa´s first COVID-19 case was recorded in Egypt on 14^th^ February 2020 and many other African countries begun registering cases through travelers returning from hotspots in Asia, Europe and the United States [3]. Uganda reported its first case on the 21^st^ March 2020, a traveler, on an international flight at Entebbe International Airport [4]. The Government of Uganda (GoU) responded by instituting multiple strategies to curb the spread of COVID-19, but the number of cases reached 6,468, with 63 deaths and 2,731 recoveries by 22^nd^ September 2020 [5].

Development of COVID-19 symptoms has been documented to occur between 2 to 14 days in a number of studies across the globe [5]. The average incubation period for COVID-19 is 5 to 6 days, however, it could go up to 14 days [6]. Lauer *et al*. (2020) explains that the incubation period can inform several important public health activities for infectious diseases, including active monitoring, surveillance, control and modeling. Imperial evidence based on 88 confirmed cases in Chinese provinces outside Wuhan showed similar findings regarding the mean incubation period of 6.4 days (95% CI, 5.6 to 7.7 days). Further, Linton *et al*. (2020) study based on 158 confirmed cases outside Wuhan established similar findings regarding timing of symptom onset documenting a median incubation period of 5 days (95% CI, 4.4 to 5.6 days), with a range of 2 to 14 days [7]. The length of active monitoring needed to limit the risk of contracting SARS-CoV-2 infection is vital in curtailing direct and indirect transmission of COVID-19.

Transmission of SARS-CoV-2 from a pre-symptomatic case can occur before symptom onset, with some cases remaining asymptomatic and others developing mild to severe symptoms [8]. In symptomatic persons, transmission occurs when non-infected persons get in direct contact through respiratory droplets or by contact with contaminated objects and surfaces [6,9]. The time of symptom onset is critical as one may be more contagious when compared to later stage of the disease [10].

While there seems to be a consensus regarding the incubation period for COVID-19 ranging between 2 to 14 days, the questions are empirical evidence on the timing of particular signs and symptoms of COVID-19 along the period and timing of symptom onset for various characteristics of cases. In COVID-19 surveillance, Uganda monitored cases and contacts for signs and symptoms of COVID-19 and samples were taken as a procedural requirement. In addition, data variables collected included age, sex, date of illness onset, date of sample taken, test result and location of testing site [10]. This data is important for monitoring and managing cases. The administrative data was entered and maintained in a national database owned by the ministry of health. Analysis of this data is critical in informing epidemiological categorization, including identification of clinical features of cases along and after the incubation period of COVID-19. This guides policy makers and public health experts in implementation of COVID-19 interventions. Using laboratory confirmed symptomatic cases of COVID-19 from the national database, our study provides empirical evidence concerning the incubation period particularly focusing on timing of symptom onset.

## Methods

We obtained administrative data from the national database owned by the ministry of health on publicly confirmed cases in Uganda who developed symptoms of COVID-19 to investigate the timing of symptom onset. The database contains all reported cases of COVID-19 from the 1^st^ confirmed case to the time of data abstraction. The database is managed by a team of experts from the incident management team of the national COVID-19 response the signs and symptoms considered were: fever, cough, sore throat, shortness of breath, headache, chest pain, running nose, nausea, vomiting, abdominal pain, muscle pain and joint pain, diarrhea, chills and general body weakness. In addition to the signs and symptoms, we obtained demographic age and sex characteristics. This is because studies on COVID-19 patients have shown that higher risk of development to the severe form of the disease and fatalities is more likely among men when compared to women [11-15]. It is important to note that our study does not make any inferences about asymptomatic infection with SARS-CoV-2. The cases were laboratory confirmed for the COVID-19 infection by real-time RT-PCR between March 21^st^ 2020 and September 4^th^ 2020. All the cases were monitored for any indication of signs and symptoms of COVID-19 before and after specimens were taken to ascertain their status [16,17]. For the purpose of this study, we utilized only 420 cases that presented at least a sign or symptom of COVID-19. The symptomatic cases represent 32.6% of the overall number of complete cases in the dataset (n=1,288). The least common symptoms were excluded from the analysis in the subsequent sections. This is because numbers of individuals who presented with the symptoms were relatively small (n≤25) for any meaningful statistical inference to be obtained from the data.

**Variables and their measurements:** timing of symptom onset for COVID-19 was considered as the outcome variable. We defined timing of onset of symptoms as a period between date when case was confirmed positive for COVID-19 using laboratory test results to the date of clinical onset of any signs and symptom. The variable denotes the period between date when case was confirmed positive for COVID-19 using laboratory test results to the date of clinical onset of any signs and symptom. Overall, the period ranges between 0 to 14 days. The number of days each of the three categories adopted in investigating the timing of symptom onset were: 0 to 3 days in the first tertile, 4 to 6 days in the 2^nd^ tertile and at least 7 days in the 3^rd^ tertile. Time zero (0) denotes cases that were clinically identified symptomatic on the same day when they were confirmed positive for the SARS-CoV-2 virus. Based on the dearth of clinically available documented evidence on how the incubation period could be appropriately categorized, we automatically generated three quantile categories using the *xtile* command in STATA 15.0. Each of the two points that divide the distribution (i.e. the incubation period) into three parts, each containing a third of the population, is referred to as a tertile. It is evident that the incubation period was investigated in our study using a normal outcome i.e. symptom onset occurred either in 1^st^ tertile, 2^nd^ tertile or 3^rd^ tertile. This is because it is highly likely that the time for symptom onset would statistically be an interval rather than a point estimate. This background forms the basis behind using a categorical outcome to establish the period or days within which particular symptoms would occur. The explanatory variables were: i) status of onset of symptoms for COVID-19 namely cough, sore throat, shortness of breath, headache, chest pain, running nose, nausea, vomiting, abdominal pain, muscle pain and joint pain, diarrhea, chills and general body weakness; ii) demographic characteristics of cases namely age and sex.

**Data analysis:** the analysis was carried out using STATA 15.0 at three stages: first, a descriptive summary of the demographic characteristics of cases (age, and sex), status of symptoms of COVID-19 and timing of onset of first symptoms was done using frequency distributions. Second, differentials in timing of symptom onset were assessed by characteristics of cases and status of onset of symptoms of COVID-19 using cross-tabular analysis. Associations were established using the Pearson Chi-square test. For the purpose of this paper, all variables that presented a relatively low probability value following the Chi-square test (p<0.5) were considered for further analysis unless otherwise stated. Third, the timing of symptom onset was investigated at the multivariable stage using a multinomial logistic regression (MLR). The model investigated the risk of symptom onset during: i) first tertile (0 to 3 days) rather than 3^rd^ tertile (beyond seven days) after laboratory confirmation for COVID-19; (ii) second tertile (4 to 7 days) rather than 3^rd^ tertile (beyond seven days) after laboratory confirmation for COVID-19. The base category in the MLR was 3^rd^ tertile i.e. beyond seven days of laboratory confirmation for COVID-19. In addition to the symptoms for COVID-19, we utilized demographic characteristics of cases namely age and sex. The MLR model was assessed for appropriateness using model fit statistics obtained via the fitstat command [12,13]. In particular, we utilized the Akaike information criterion (AIC) and Bayesian information criterion (BIC) to compare different possible models and determine which one was the best fit for the data. A lower AIC or BIC value indicated better fit - model that explains the greatest amount of variation using the fewest possible independent variables.

**Limitations of the study:** similar to the challenges of administrative data elsewhere, the data had missing information. Thus, we utilized data with complete information for the variables of interest to address the issue. In addition, we did some data cleaning to correct any internal inconsistences that existed in the data. For example, some cases had erroneously been regarded as asymptomatic yet a date of clinical onset of symptoms and presentation of signs and symptoms were captured in the database.

## Results

**Characteristics of symptomatic cases:** by September 4^th^ 2020, a total of 1,288 complete records of publicly reported confirmed cases for SARS-CoV-2 infection were available in the dataset. A total of 420 out of the 1,288 cases were symptomatic. Case patients were predominantly male (72.4%), median age was 33 years (interquartile range (IQR)=26-41); the highest proposition were in their thirties with regard to age (36.4%), followed by those aged 20 to 29 (25.1%) and 40 to 49 (14.9%) while the rest were either above 49 (13.0%) or below 20 (10.6%) years (Table 1).

**Table 1:** demographic characteristics of cases assessed in the study

Characteristics	Frequency (n=420)	Percentage (%)
**Age**		
19 below	44	10.6
20-29	104	25.1
30-39	151	36.4
40-49	62	14.9
50 above	54	13.0
Total	415	100.0
**Sex**		
Male	304	72.4
Female	116	27.6
Total	420	100.0

**Figure 1:**
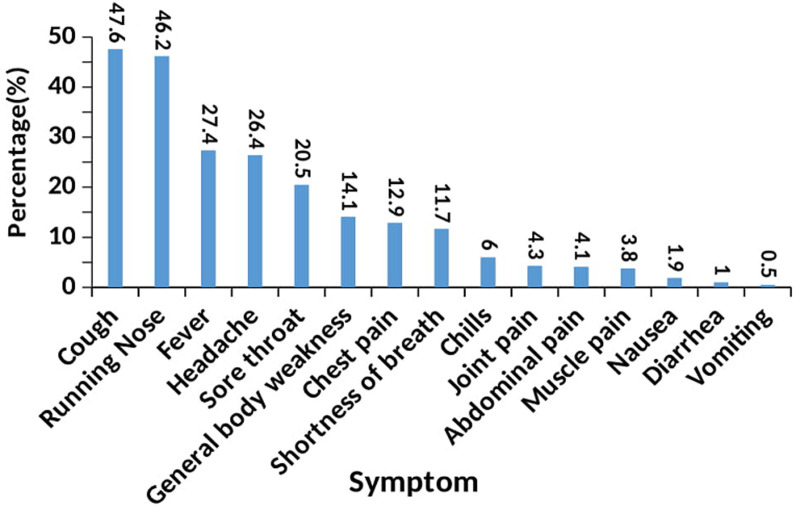
distribution by clinical onset of first symptoms for COVID-19 in Uganda

**Onset of symptoms for COVID-19:** the commonest symptoms among the 420 case patients who presented with symptoms were: cough (47.6%), running nose (46.2%), fever (27.4%) as well as headache (26.4%) and sore throat (20.5%). The least common symptoms were vomiting (0.5%), diarrhea (1.0%), nausea (1.9%) as well as muscle pain (3.8), abdominal pain (4.1%) and joint pain (4.3%) (Figure 1).

**Figure 2:**
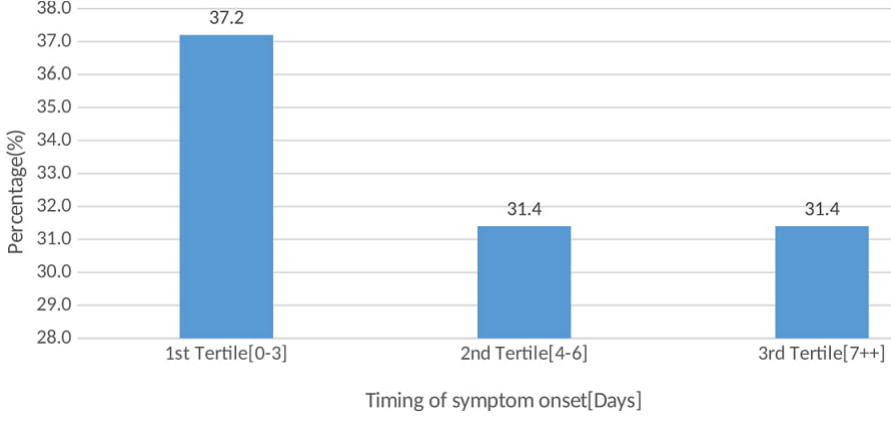
timing of onset of first symptom(s) for COVID-19

**Timing of symptom onset for COVID-19:** despite the timing for symptom onset being treated as a nominal outcome using three categories, it is important to note that median time to symptom onset was 5.0 days (range, 0 - 14 days, IQR=2-8). The distribution of the time to symptom onset is presented in Figure 2. The illustration shows that the highest proportion of cases (37.5%) presented symptom(s) within the three days after laboratory confirmation for the SARS-CoV-2. The rest presented symptoms either in the 2^nd^ tertile (31.4%) denoting 4 to 6 days or 3^rd^ tertile (31.4%) denoting at least 7 days after being confirmed positive for SARS-CoV-2.

**Differentials in timing of symptom onset:** Table 2 presents a cross-tabular analysis of differentials in timing of symptom onset. It is important to note that only cases where a symptom is presented are shown in the table. In the results, the common symptoms in the 1^st^ tertile were: sore throat (44.6%), fever (41.2%), shortness of breath (36.4%) and cough (34.9%). Running nose (33.9%) was the common symptom in the 2^nd^ tertile denoting 4 to 6 days after exposure to the virus. The common symptom in the 3^rd^ tertile, beyond six days after laboratory confirmation for COVID-19, were chest pain (51.2%), general body weakness (43.9%), headache (39.7%) and cough (37.1%) (Table 2).

**Table 2:** differentials on timing of clinical onset of symptoms for COVID-19

Characteristics of cases	Frequency	Timing of symptom onset (%)			Chi^2^	p-value
		First tertile	Second tertile	Third tertile		
**Demographics**						
**Sex**						
Male	215	37.2	30.7	32.1	0.250	0.882
Female	78	37.2	33.3	29.5		
**Age group**						
19 Below	35	42.9	48.6	8.6	14.60	0.067
20-29	74	37.8	31.1	31.1		
30-39	101	34.7	30.7	34.7		
40-49	41	41.5	29.3	29.3		
50 above	39	33.3	20.5	46.2		
**Signs and symptoms**						
Fever	89	41.2	23.5	35.3	3.44	0.148
Cough	145	34.9	28.0	37.1	3.73	0.155
Sore throat	56	44.6	23.2	32.1	2.51	0.284
Shortness of breath	33	36.4	27.3	36.4	0.50	0.779
Headache	68	27.9	32.4	39.7	4.01	0.134
Chest pain	41	31.7	17.1	51.2	9.46	0.009
Running nose	130	30.0	33.9	36.2	5.38	0.068
General body weakness	41	31.7	24.4	43.9	3.50	0.173

**Predicting timing of symptom onset:** the risk of symptom onset in the 1^st^ tertile, rather than in the 3^rd^ tertile, was significantly associated with age and running nose (p<0.05). These findings can be explained as follows: cases aged 20 to 29 (RRR=0.21, 95% CI 0.05-0.87), 30 to 39 (RRR=0.20, 95% CI 0.05 - 0.81) and above 49 (RRR=0.13, 95% CI 0.02 - 0.61) were less likely to have symptoms in the 1^st^ tertile rather than in the 3^rd^ tertile compared to those aged below 20 years. Cases with running nose were less likely to present the symptom within the 1^st^ tertile rather than in the 3^rd^ tertile compared to those without (RRR=0.45, 95% CI 0.24 - 0.84). Although marginally significant, it is worth noting that headache (p=0.065) and chest pain (p=0.052) were less likely to present in the 1^st^ tertile rather than the 3^rd^ tertile.

The risk of symptom onset within the 2^nd^ tertile, rather than in the 3^rd^ tertile, was significantly associated with age and chest pain (p<0.05). These findings can be explained as follows: cases aged 20 to 29 years (RRR=0.20, 95% CI 0.04 - 0.83), 30 to 39 years (RRR=0.18, 95% CI 0.04 - 0.72), 40 to 49 years (RRR=0.20, 95% CI 0.04 - 0.92) and above 49 years (RRR=0.09, 95% CI 0.01 - 0.47) were less likely to have symptoms in the 2^nd^ tertile rather than in the 3^rd^ tertile compared to those aged below 20 years. Chest pain was less likely to present within the 2^nd^ tertile rather than in the 3^rd^ tertile compared (RRR=0.26, 95% CI 0.09 - 0.72). Although marginally significant, it is worth noting that cough (p=0.051) was less likely to present in the 2^nd^ tertile rather than the 3^rd^ tertile (Table 3).

**Table 3:** predicting timing of clinical onset of signs and symptoms for COVID-19

Symptoms	First tertile (0-3)			Second tertile (4-7)		
	RRR (95% CI)	Std. Err	p-value	RRR (95% CI)	Std. Err	p-value
**Sex**						
Male	1.00	.	.	1.00	.	.
Female	0.91 (0.46 - 1.81)	0.319	0.809	0.89 (0.43 - 1.82)	0.326	0.761
**Age**						
19 below	1.00	.	.	1.00	.	.
20-29	0.21 (0.05 - 0.87)	0.153	0.032	0.20 (0.04 - 0.83)	0.146	0.027
30-39	0.20 (0.05 - 0.81)	0.145	0.025	0.18 (0.04 - 0.72)	0.129	0.015
40-49	0.25 (0.05 - 1.16)	0.198	0.078	0.20 (0.04 - 0.92)	0.156	0.039
50+	0.13 (0.02 - 0.61)	0.102	0.010	0.09 (0.01 - 0.47)	0.077	0.004
**Fever**						
No	1.00	.	.	1.00	.	.
Yes	0.92 (0.48 - 1.78)	0.309	0.824	0.55 (0.26 - 1.15)	0.206	0.114
**Cough**						
No	1.00	.	.	1.00	.	.
Yes	0.62 (0.34 - 1.15)	0.194	0.133	0.53 (0.28 - 1.00)	0.172	0.051
**Sore throat**						
No	1.00	.	.	1.00	.	.
Yes	1.32 (0.63 - 2.77)	0.498	0.453	0.70 (0.30 - 1.65)	0.306	0.425
**Shortness of breath**						
No	1.00	.	.	1.00	.	.
Yes	1.23 (0.44 - 3.47)	0.652	0.683	1.59 (0.51 - 4.90)	0.913	0.414
**Headache**						
No	1.00	.	.	1.00	.	.
Yes	0.50 (0.24 - 1.04)	0.187	0.065	0.78 (0.37 - 1.62)	0.291	0.508
**Chest pain**						
No	1.00	.	.	1.00	.	.
Yes	0.43 (0.18 - 1.00)	0.186	0.052	0.26 (0.09 - 0.72)	0.135	0.009
**Running Nose**						
No	1.00	.	.	1.00	.	.
Yes	0.45 (0.24 - 0.84)	0.142	0.012	0.64 (0.33 - 1.21)	0.209	0.176
	12.46 (2.95 - 52.50)	9.14	0.001	13.36 (3.15 - 56.53)	9.83	0.000

## Discussion

The common symptoms at onset of illness in the findings were cough, running nose, fever, headache and sore throat; while the less common symptoms were vomiting, diarrhea, nausea as well as abdominal pain and joint pain. The highest proportion of cases (37.5%) presented symptom in the 1^st^ tertile denoting onset within three days after laboratory confirmation for SARS-CoV-2; while the rest had symptoms either in the 2^nd^ (31.4%) or 3^rd^ tertile (31.4%) denoting 4 to 6 days and at least 7 days after exposure, respectively. Running nose and chest pain were less likely to occur in the 1^st^ tertile (relative risk ratio (RRR)=0.45, 95%CI 0.24 - 0.84) and 2^nd^ tertile (RRR=0.64, 95%CI 0.09 - 0.72), than in the 3^rd^ tertile. Cases aged 20 to 29, 30 to 39, 40 to 49 and above 49 years were less likely to have symptoms in the 1^st^ and 2^nd^ tertile compared to those aged below 20 years (p<0.05).

The clinical features observed in Uganda were features in in Wuhan, China. These were: fever, cough and myalgia or fatigue while the less common symptoms were sputum production, headache, haemoptysis and diarrhoea [18,19]. Both studies present cough and fever as common symptoms at onset of illness and diarrhea as the less common symptom. However, headache was established as one of the common symptoms at onset among patients in Uganda while it was established among the less common symptoms in Wuhan, China. Further, muscle and joint pain were established as less common symptoms at onset in Uganda while these were established among the common symptoms in Wuhan, China. This evidence shows both similarities and differences in epidemiological characterization of cases exposed to COVID-19 in Uganda and elsewhere. Chest pain which occurred most in 3^rd^ tertile is considered as one of the emergency warning signs and symptoms for COVID-19 [20]. Because it occurred in the 3^rd^ tertile, it´s an indication of disease progression from mild to severe form of COVID-19 illness.

Symptom onset in the 1^st^ and 2^nd^ tertile was less likely among cases aged 20 years and above compared to the younger ones. This implies that younger persons were more likely to have symptom onset earlier after exposure to COVID-19 compared to their older counterparts. Though the younger age group onset of symptoms occurred earlier than the older age groups, the symptoms were mild rather than severe. Our findings concur with evidence in literature that older people are at a higher risk of getting severe COVID-19 disease [20]. It´s important to note that our study population were predominantly a young population (median age is 33 years, IQR=26 - 41). The younger population with mild disease should call for vigilance is surveillance because, they can be a source of community spread of infection and a risk to the older generation in their localities. Our study showed no significant variations in timing of onset for the following symptoms: fever, sore throat, shortness of breath, cough and headache (p>0.05).

Most studies have shown that majority of confirmed cases for the SARS-CoV-2 infection are males [21-23]. We however, found no significant variation in timing of symptom onset by sex (p>0.05). This evidence seems contrary to studies in China, South Korea, United States and Italy that have reported an association between the sex of COVID-19 patients and fatality rates as well as critically ill status, with males have higher mortality rates [21-23]. Less female patients also needed intensive care compared to male patients and some symptoms were experienced more by males than females, including cough and fever [24]. In a study of sex differentials in COVID-19 patients, Asghar *et al*. arguments for these variations include the following: women´s immune cells activate more than men, women produce lower levels of interleukin-6 (IL-6) compared to men, which is associated with better longevity; different levels of angiotensin converting enzyme-2 (ACE2) in men and women, the effects of testosterone on ACE2 levels and the fact that the ACE2 gene is located on the X-chromosome. In citing Fagone *et al*. [25], Maleki *et al*. writes: “in age group of 40 to 60 years, the transcriptomic characteristics of female lung tissue has more similarities to COVID-19 induced characteristics compared to male tissue. A possible explanation of the lower incidence of COVID-19 in female patients could be the lower threshold of acute immune response to COVID-19 in men when compared to women”.

## Conclusion

Studies have shown that the time between exposure to the virus and symptom onset can be up to 14 days. Our study characterized the period within three groups namely 1^st^ tertile, 2^nd^ tertile and 3^rd^ tertile. Running nose and chest pain were less likely to occur in the 1^st^ and 2^nd^ tertile, denoting 0 to 3 days and 4 to 6 days after laboratory confirmed for COVID-19 infection, respectively. Cases aged 20 years and above were less likely to present symptoms in the 1^st^ and 2^nd^ tertile. Our study provides empirical evidence for epidemiological characterization of cases by signs and symptoms along the incubation period. This complements the current proposals for the length of active monitoring of persons potentially exposed to SARS-CoV-2. It is important to note that publicly reported cases may over represent severe cases, the incubation period for which may differ from that of mild cases. In addition, our study does not assess any co-morbidities which may potentially influence timing of symptom onset. Therefore, our findings should be interpreted in light of this consideration.

### What is known about this topic

- The incubation period is well documented amongst Caucasians and Orientals;
- Cough is the earliest symptom to occur;
- The variations of incubation period determined and varied greatly.

### What this study adds

- The timing of symptom onset of COVID-19 among Africans in the tropics;
- The age difference in occurrence of symptoms;
- Characterization of patients using symptoms.
